# Promoting weight loss through diet and exercise in overweight or obese breast cancer survivors (InForma): study protocol for a randomized controlled trial

**DOI:** 10.1186/s13063-016-1487-x

**Published:** 2016-07-28

**Authors:** Patrizia Gnagnarella, Daniele Dragà, Federica Baggi, Maria Claudia Simoncini, Annarita Sabbatini, Ketti Mazzocco, Fabio Domenico Bassi, Gabriella Pravettoni, Patrick Maisonneuve

**Affiliations:** 1 Division of Epidemiology and Biostatistics, European Institute of Oncology, Via Adamello, 16, 20139 Milan, Italy; 2 Physiotherapy Unit, European Institute of Oncology, Via Ripamonti 435, 20141 Milan, Italy; 3 Dietetic and Clinical Nutrition Unit, European Institute of Oncology, Via Ripamonti, 435, 20141 Milan, Italy; 4 Department of Oncology and Hemato-oncology, University of Milan, Via Festa del Perdono, 7, 20122 Milan, Italy; 5 Applied Research Division for Cognitive and Psychological Sciences, European Institute of Oncology, Via Ripamonti, 435, 20141 Milan, Italy; 6 Division of Breast Surgery, European Institute of Oncology, Via Ripamonti, 435, 20141 Milan, Italy

**Keywords:** Breast cancer survivors, Randomized controlled trial, Weight loss, Diet, Physical activity, Prevention, Counseling, Pedometer, Quality of life, Body composition

## Abstract

**Background:**

Most women with breast cancer experience a progressive weight gain during and after treatment. Obesity is associated with an increased risk of recurrence, contralateral breast cancer, and death. Physical activity after cancer diagnosis has been reported to have positive effects on body composition and quality of life. We present the protocol of the InForma study, a trial testing the efficacy of an intervention on weight loss (≥5 % of the baseline body weight) in a group of overweight or obese breast cancer survivors.

**Methods/design:**

This is a four-arm randomized controlled trial. Patients will receive a 6-month intervention and be followed for a further 18 months. Intervention is designed to improve adherence to a healthy diet and/or to increase physical activity, taking advantage of a wrist-based activity monitor. Participants will be recruited among overweight or obese breast cancer patients treated at the European Institute of Oncology, after completion of eventual adjuvant chemotherapy and/or radiotherapy. It is envisaged that 260 patients will be randomized into four arms: Dietary Intervention; Physical Activity Intervention; Physical Activity and Dietary Intervention; and Less Intensive Intervention. Women will be offered individualized counseling consisting of face-to face discussion and phone calls in addition to group meetings. A motivational interviewing approach will be used to encourage health behavior change. All participants will be given a pedometer device to monitor their physical activity. Participants’ dietary intake will be repeatedly assessed using a validated food frequency questionnaire. Participants’ quality of life and anxiety will be assessed with the Functional Assessment of Cancer Therapy-Breast and the State-Trait Anxiety Inventory questionnaires. Blood samples will be collected at baseline and follow-up visits to assess lipid and hormone profiles. Body composition will be repeatedly assessed using bioelectrical impedance vector analysis for identifying changes of fat and fat-free mass. Women allocated to the less intensive intervention arm will be considered as the control group.

**Discussion:**

While there is a rising concern about the role of obesity in cancer recurrence and survival, this trial with its multi-arm design, motivational approach and use of a pedometer device will provide important insights regarding the most effective approach in promoting weight control in breast cancer survivors.

**Trial registration:**

ISRCTN53325751 (registration date: 16 October 2015); ClinicalTrials.gov NCT02622711 (registration date: 2 December 2015).

## Background

Breast cancer is the most frequently diagnosed form of cancer and the leading cause of cancer death among women worldwide, with an estimated 1.7 million new cases and 521,900 deaths in 2012 [1]. According to the association of Italian Cancer Registries [2], breast cancer is also the most common cancer in women in Italy with 581,373 newly diagnosed cases in 2010. The estimated incidence is stable and mortality is decreasing mainly due to advances in diagnosis and treatment. Consequently survival from breast cancer has improved, and currently about 87 % of breast cancer patients in Italy survive more than 5 years [2].

Modifiable lifestyle factors such as obesity, physical inactivity and excessive alcohol consumption are established risk factors for the development of, and death from, breast cancer [3, 4]. There is also accepted evidence that, after a diagnosis of breast cancer, being overweight or obese is associated with an increased risk of breast cancer relapse, death [5, 6], or second primary contralateral breast cancer [7–9]. In a recent meta-analysis including more than 200,000 patients with breast cancer, Chan et al. [10] demonstrated a 41 % increase in overall mortality in obese and 7 % in overweight women and a 35 % increase in breast cancer-specific mortality in obese women and 11 % in overweight women, as compared with patients who had normal weight at diagnosis.

Most women experience a progressive weight gain during and after treatment for breast cancer [11, 12] due to the changes in hormone levels and body composition associated with treatment. Weight gain usually ranges from 2–6 kg in the first year following diagnosis, but greater gains are also common [13]. Chemotherapy is associated with weight gain due to a reduced metabolism and the subsequent induction of menopause increasing the likelihood of weight gain [13]. Moreover treatment-related side effects, duration of treatments and psychological distress may affect diet and physical activity patterns compromising energy balance in ways that promote weight gain.

High body mass index (BMI) results in an increased production of hormones (estrogens, insulin, leptin), and pro-inflammatory cytokines, which have, in turn, been associated with breast cancer risk [14, 15]. In fact, estrogenic stimulation promotes breast cancer pathogenesis and tumorgenesis, while insulin and leptin exhibit proliferative, mitogenic, and anti-apoptotic activities in mammary cells, thus promoting tumor growth [16]. High circulating estrogen levels are associated with increased risk of breast cancer recurrence [17, 18].

Alongside this, physical inactivity represents a leading cause of death worldwide [19]. Regular physical activity yields a wide range of health benefits, because it is associated with healthier levels of circulating hormones (insulin, leptin and insulin-like growth factor (IGF)-1 and IGF-3, estrogens and androgens) [20, 21] as well as possibly strengthening the immune system [22]. Moreover physical activity after breast cancer diagnosis has also been reported to have positive effects on body composition, psychological outcomes, and quality of life (QoL) [23].

The number of trials of weight loss interventions in breast cancer is increasing, but a demonstration of their benefit on breast cancer outcome is limited [24]. Weight loss resulting from intentional increased physical activity and caloric restriction can improve hormonal status, quality of life, and physical functioning among obese and overweight breast cancer survivors [9, 24, 25]. Regular physical activity and healthy dietary habits are important factors for reducing the risk of cancer recurrence and mortality, as well as lifestyle-related chronic conditions [7, 26]. Few intervention trials in breast cancer survivors, integrating physical activity and dietary intervention have been initiated or conducted so far [24, 27, 28].

Despite the accumulating evidence of the detrimental effect of physical inactivity and being overweight on breast cancer risk and risk of breast cancer recurrence, weight control and physical activity counseling are not yet current practice. Clinicians typically try to advise patients to change their lifestyle habits using a directing style but this approach is often ineffective. Patients often seem ambivalent or unmotivated in respect to these messages and, in turn, generate resistance or passivity. Motivational interviewing is an alternative approach to discussing behavior change that fosters a constructive specialist-patient relationship and leads to better outcomes for patients. This intervention, aimed at encouraging positive health behavior changes within a medical setting, is promising [29], and it can help individuals to enhance adherence to diet and exercise modification programs [30].

The prognostic role of body mass index has been previously assessed on the outcome of a consecutive series of women treated at our institution (the European Institute of Oncology (IEO), Milan, Italy) for an aggressive form [estrogen receptor/human epidermal growth factor receptor 2 (ER-/HER2)-positive] of breast cancer. In these patients, obesity was significantly associated with the risk of developing distant metastases and with worse overall survival [31].

Given the above considerations, our aim is to evaluate the effect of a 6-month intervention on weight loss in a group of overweight or obese women treated for early breast cancer. The intervention is designed to improve adherence to a healthy diet or/and to increase physical activity and decreased sedentary time, taking advantage of a wrist-based activity monitor (pedometer device).

## Methods/design

### Study design

**Fig. 1:**
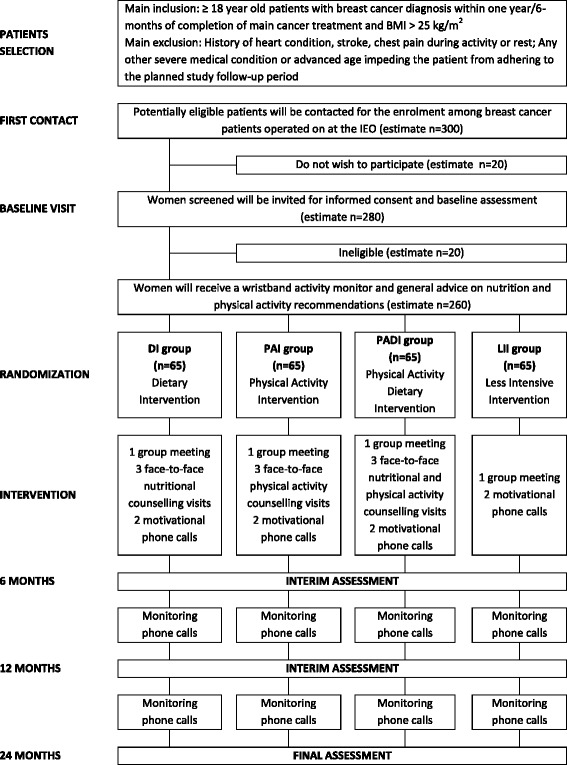
Study flowchart

The present study (InForma) was designed as a mono-institutional randomized controlled four-arm parallel-group trial. Randomized patients will receive a 6-month intervention and will be followed for further 18 months (Fig. 1).

### Study population and randomization

Potential study participants will be recruited among all overweight or obese breast cancer patients treated at the IEO after breast cancer treatments (surgery, chemotherapy and/or radiation therapy) have been completed. Women undergoing hormone therapy will be eligible for the study. Groups of eligible participants will be invited at IEO for further eligibility assessment and enrolment. On arrival, they will receive a progressive number from the front desk receptionist. After an information meeting, they will be invited to sign all the study documents (informed consent form, privacy disclosure). Height, weight, waist and hip circumferences, and body composition will be measured. Participants who are eligible will receive the study questionnaires to be filled in. They will then be called following their random arrival number and be allocated in a 1:1:1:1 ratio to one of the four intervention arms: Dietary Intervention (DI); Physical Activity Intervention (PAI); Physical Activity and Dietary Intervention (PADI); or Less Intensive Intervention (LII) following a pre-established randomization list (Fig. 1). The randomization list was created using a computer random number generator and securely kept by a study collaborator not involved at the front desk. Randomization is not stratified, but blocks of 20 were created to achieve balance in the allocation of participants to the four treatment arms. The randomization list is not known to the front desk receptionist (administrative staff) who provides the progressive number that will be matched with the randomization number at the end of the information meeting. Randomization will be made by a member of the InForma study team (dietician or physiotherapist). This to ensure that allocation cannot be guessed before registration. Allocation is fixed and cannot be changed after randomization.

### Study outcomes

The main aim of the study is to evaluate the impact of a 6-month intervention program on body weight change (weight loss ≥5 % of the baseline body weight) in overweight or obese breast cancer survivors.

As secondary outcomes, we will further examine the impact of the intervention on long-term weight control or maintenance, physical activity and inactivity levels, dietary intake, health status, and quality of life. We will also examine the effect of intervention on blood lipid profile [total, high-density lipoprotein (HDL), low-density lipoprotein (LDL) and very low density lipoprotein (VLDL) cholesterol and triglycerides] and on estradiol, C-reactive protein (CRP), insulin, and glucose at 6-, 12- and 24-month visits.

### Inclusion criteria

The study will be open to overweight or obese (BMI > 25 kg/m2) women, aged 18 years or older, who have been treated for a histologically confirmed, invasive, non-metastatic breast cancer and have completed their main cancer treatment (surgery, chemotherapy, radiation therapy) for more than 6 months.

Only those women who are able to participate in the intervention, who agree to wear the wrist-based activity monitor during the study period, and who sign the informed consent form and the privacy disclosure, will be enrolled.

### Exclusion criteria

Women satisfying any of the following criteria will not be eligible for the study: previous severe medical condition(s); advanced age impeding their adherence to the planned study schedules; contraindications to exercise due to any heart condition, stroke, chest pain during activity or rest; severe hypertension and any orthopedic complications that would prevent optimal participation in the physical activities prescribed; unable to walk for exercise (self-reported); symptoms of alcohol or substance dependence.

Other non-medical exclusion criteria are: lack of transportation to the study location over the study period; plans to move away from Lombardy or to be inaccessible for more than 3 weeks during the intervention period.

### Intervention

The principal aim of the intervention is to promote weight loss through diet and physical activity alone or in combination. The target goal is a 5 % weight loss 6 months after randomization. Participants will be followed up for a further 18 months and secondary outcome assessments will be made at 12 and 24 months (Table 1).

**Table 1:** Study assessments

Assessments	Instrument	Visits			
		Baseline	6-month	12-month	24-month
Total, HDL, LDL and VLDL cholesterol, triglycerides, estradiol, insulin, glucose and C-reactive protein	Blood test	√	√		√
Height^a^, weight, waist and hip circumference, body mass index, waist-to-hip ratio	Calibrated scales, stadiometer, tape measure	√	√	√	√
Body composition (including total body water (TBW), body cell mass (BCM), extracellular water (ECW), fat-free mass (FFM), fat mass (FM) and percentage fat mass (% FM)	Bioelectrical impedance vector analysis (BIVA) (Nutrilab device, AKERN Srl – Italy)	√	√	√	√
Dietary target	24 hr recalls and/or food diary	√^b^	√		
Steps	Pedometer-like device	√^b^	√		
Diet	Food Frequency Questionnaire (FFQ)	√	√		√
Quality of life	Functional Assessment of Cancer Therapy- Breast (FACT-B)	√	√		√
Mood	State-Trait Anxiety Inventory (STAI)	√	√		√
Physical activity	International Physical Activity Questionnaire (IPAQ)	√	√		√

^a^Height will be assessed only at baseline

^b^24 hr recalls and/or food diary and the pedometer-like device will be used during the intervention to monitor individualized goals

Baseline assessment will be made for all participating women, prior to randomization in one of the four intervention arms (DI, PAI, PADI, and LII). All participants must wear the wrist-based activity monitor during the 6-month intervention period (day and night).

Participants randomized to one of the three intervention arms (DI, PAI, and PADI) will be given individualized counseling based on their lifestyle habits to reduce their weight. The motivational interviewing approach will be used to discuss behavior change that fosters a constructive specialist-patient relationship which could lead to better body weight control for the patients.

The intervention will begin with an intensive phase consisting of monthly contacts: three face-to-face meetings, one group meeting and two motivational phone calls. The group sessions are planned for closed groups of an average of ten women per arm and will consist of a motivation and information session to discuss strategies and to reinforce guidance. The participants may invite a caregiver or a relative during the group meeting. The meeting aims to offer a caring and supportive environment where its members support each other and explore new strategies to create long-lasting positive changes. At baseline, their requirement for weight maintenance will be calculated using the Schofield equation [32] and then multiplied by their current physical activity level before applying a caloric restriction of about 500–1000 kilocalorie (kcal)/day [33, 34].

Individualized goals will be set for all participants included in the three intervention arms to promote weight loss. These goals will be verified at each contact and workable solutions will be proposed in case of specific problems. The contents of all contacts are presented in Table 2.

**Table 2:** Delivery and contents of the 6-month intervention visits for participants randomized to the intervention arms: Dietary Intervention (DI); Physical Activity Intervention (PAI); Physical Activity; and Dietary Intervention (PADI)

	Contact 1	Contact 2	Contact 3	Contact 4	Contact 5	Contact 6
Contact	Face-to-face	Telephone	Group meeting	Face-to-face	Telephone	Face-to-face
Time line	1 month (following baseline assessment)	2 months	3 months	4 months	5 months	6 months
Duration	30 min	15 min	90 min	30 min	15 min	30 min
Who delivers	Dietician and/or physiotherapist	Dietician and/or physiotherapist	Psychologist, dietician and physiotherapist	Dietician and/or physiotherapist	Dietician and/or physiotherapist	Dietician and/or physiotherapist
Social support	-	-	Invited friend/partner/family member	-	-	-
Motivational approaches	1/2 week diet/activity diaries assessment	Identify perceived diet/activity challenges	Coping and provide opportunities to learn from each other	1/2 week diet/activity diaries assessment	Identify perceived diet/activity challenges	1/2 week diet/activity diaries assessment
	Identify perceived diet/activity challenges	Provide feedback and encouragement	Review and discuss members’ goal evaluations	Identify perceived diet/activity challenges	Provide feedback and encouragement	Identify perceived diet/activity challenges
	Identify motivations, confidence, ambivalence regarding lifestyle change	Revaluate motivations, confidence, ambivalence regarding lifestyle change	Evaluate and discuss members’ motivation to change	Provide feedback and encouragement Revaluate motivations, confidence, ambivalence regarding lifestyle change	Revaluate motivations, confidence, ambivalence regarding life style change	Provide feedback and encouragement Revaluate motivations, confidence, ambivalence regarding lifestyle change
Informing change	Healthy eating and activity principles Portion size	Discuss experience of changing diet and activity referring back to perceived challenges at previous visit.	Provide general advice for psychological well-being and health promotion	Discuss experience of changing diet and activity referring back to perceived challenges at previous visit.	Discuss experience of changing diet and activity referring back to perceived challenges at previous visit.	Discuss experience of changing diet and activity referring back to perceived challenges at previous visit.
	Energy-dense food and drinks Activity and inactivity	Check for queries or areas of confusion or elaboration		Check for queries or areas of confusion or elaboration	Check for queries or areas of confusion or elaboration	Check for queries or areas of confusion or elaboration
	Discuss pedometer data	Discuss pedometer data		Discuss pedometer data	Discuss pedometer data	Discuss pedometer data
Personal goals	Setting diet or activity personal goals	Discuss self-monitoring of personal goals	-	Discuss self-monitoring of personal goals	Discuss self-monitoring of personal goals	Discuss self-monitoring of personal goals
		Provide positive feedback		Provide positive feedback	Provide positive feedback	Provide positive feedback
		Identify new personal goals or redefine old ones		Identify new personal goals or redefine old ones	Identify new personal goals or redefine old ones	Negotiate key long-term diet and activity goals based on: perceived achievements
						Summarize success
	Identify next appointment	Confirm group meeting	Identify date for next appointment	Identify date for motivation call	Identify last appointment	Identify date for motivation call

From 6 months onward, participants will be monitored by phone calls during which the strategies and guidance discussed in the face-to-face sessions will be repeated and reinforced.

### DI arm

The overall content of the intervention consists of an individualized diet modification proposal that will promote an energy deficit. The educational components of healthy eating habits will be based on national and international recommendations [4, 35]. Strategies and approaches to be applied include self-monitoring of food intake; realistic goal-setting, using behavior-specific goals and a step-wise approach to promote self-efficacy; addressing body image concerns and prevention of cancer relapse.

Safe weight loss should be achieved through a balanced diet according to the Italian dietary reference intakes [36]. Key strategies will emphasize the reduction of energy density by low-energy dense foods (e.g., water- and fiber-rich vegetables and fruits), limiting the intake of foods and beverages high in fat and added sugar, and limiting portion sizes.

Each goal will be stated and verified at each contact (reduce the portion size of cheese instead of normal portion size; choose fresh fish, instead of preserved fish, freshly squeezed lemon juice in water, instead of soft drinks). Participants will also be advised not to fall too far below their goal to support nutritional adequacy.

### PAI arm

The PAI will be designed on an individualized level, whereby specific activities are planned based on capabilities, lifestyle pattern and preferences so as to increase physical activity and to reduce sedentary time. Realistic and achievable goals will be set working toward short-term and long-term goals. A priority will be placed on regular planned aerobic exercise, since this creates an energy deficit that is much greater per unit of time than strength training. The general exercise prescription planned will be of low to moderate intensity, regular frequency, involving aerobic, resistance or mixed exercise types. The physical activity targets are: to be moderately physically active, equivalent to brisk walking for at least 30 min a day (short-term goal); to do purposeful exercise at a moderate level of intensity at least 60 min/day or ≥30 min of vigorous physical activity every day (long-term goal) [34, 37]. Reasonably, the initial goal will be to plan and implement daily purposeful mild to moderate exercise for a minimum of at least 10 min/day with a stepwise increase in time and intensity that will be evaluated and modified at each contact. Women who exercised at a higher level than 10 min/day at baseline will be instructed to aim for an incrementally greater goal of daily exercise and to plan to increase intensity as tolerated. Participants will wear the wrist-based activity monitor and they will be instructed to count the total number of steps to improve their self-monitoring and to reduce sedentary time.

Personalized goals will be set to increase physical activity. Each goal will be stated and verified at each contact (use public transportation to go to work instead of the car; use the stairs instead of using the elevator, check the wrist-based activity monitor). Safe increased physical activity should be recommended through a program tailored to individual characteristics. Participants will be instructed to report any problem or adverse event immediately to the clinical staff.

### PADI arm

The intervention will be focused on weight loss combining a reduced energy intake, an increase of physical activity, and reduction of physical inactivity. Women randomized to this arm will be offered an integrated version of both intervention components of the DI and PAI arms. This integrated approach will be weighted to avoid intervention overload. Individualized goals will be set for all participants to promote individualized diet modification and individualized physical activity based on individual characteristics.

### LII arm

Participants included in the LII arm will be considered as the control group. They will receive general counseling on health to reduce weight, and be provided with materials and guidelines available to the general public [4, 34, 35] and receive two motivational phone calls.

### Measures

Weight, and waist and hip circumferences will be measured at baseline, at 6-, 12-, and 24-month follow-up visits. Height will be measured at baseline. A fasting blood sample will be collected at baseline, at 6, and 24 months to measure lipid profile (total, HDL, LDL and VLDL cholesterol and triglycerides) and estradiol, insulin, glucose, and C-reactive protein level (Table 1).

Body composition will be assessed at baseline and at 6-, 12- and 24-month follow-up visits, using bioelectrical impedance vector analysis (BIVA) (Nutrilab device, AKERN Srl, Pontassieve, Italy). BIVA is a more accurate method for a quick measurement of body compartments [38, 39]. The direct analysis of the two components of the impedance vector (Z), resistance (R, Ohm), and reactance (Xc, Ohm), allows a semiquantitative evaluation of body composition in terms of body cell mass and hydration status. Data for total body water (TBW), body cell mass (BCM), extracellular water (ECW), fat-free mass (FFM), fat mass (FM) and percentage fat mass (% FM) will be available for all participants and will be used for identifying changes of fat and fat-free mass over the study period.

Physical activity will be also measured via the International Physical Activity Questionnaire (IPAQ) at baseline, 6 and 24 months. This questionnaire consists of questions that record the frequency and duration of mild, moderate, and strenuous exercise performed during free time in the previous 7 days measuring physical activity and inactivity. It is a validated self-report measure of exercise that has been reliably used in previous studies [40]. The total hours per week spent in each activity will be multiplied by the estimated metabolic cost of each activity [metabolic equivalent (MET) value] as determined from the Compendium of Physical Activities [41].

Dietary intake will be measured at baseline, 6 and 24 months using the validated and self-administered food frequency questionnaire (FFQ) developed for the European Prospective Investigation into Cancer and Nutrition Italian section (EPIC) study [42, 43]. It records daily intake of foods and nutrients over the previous year. It consists of 15 sections (first course, second course, side dish, fruit, etc.) and contains 254 questions investigating a wide range of food items.

Quality of life (QoL), health status and anxiety will be measured at baseline, 6-, 12- and 24-month visits using the Functional Assessment of Cancer Therapy-Breast (FACT-B) and State-Trait Anxiety Inventory (STAI) questionnaires. The FACT-B is a 37-item validated multidimensional self-report questionnaire, subdivided into four primary QoL domains and a disease-specific domain – additional concerns for breast cancer [44]. The subscales measure physical, social, emotional, and functional well-being, relationship with the doctor, and breast cancer concerns. The FACT-B has a 5-point Likert-type response scale. It has good psychometric properties, discriminates well between clinically distinct groups, and is responsive to change.

The STAI is a psychological inventory based two 20-item self-report measures. It assesses how respondents feel “right now, at this moment” and how respondents “generally feel”. In addition, the STAI State and Trait each have been found to contain two factors, which Spielberger labeled anxiety-present and anxiety-absent [45]. Respondents are asked to rate themselves on each item on the basis of a 4-point Likert scale, ranging from “not at all” to “very much so” for the STAI State and from “almost never” to “almost always” for the STAI Trait.

The intervention will propose the use of a pedometer-like device (a wristband) that encourages both increased physical activity and decreased sedentary time. This device is able to record total steps taken, total distance traveled, total time active during the day, and the greatest length of time of consistent movements done during the day. In addition it records light and deep sleeping time. All participants will receive and will have to wear the wrist-based activity monitor during the 6-month study period (days and nights). They could use a smartphone or a computer application to obtain feedback on their activity and sedentary time.

### Serious adverse events

We expect a very low risk for participants. We do not foresee any serious adverse events. However, if participants do experience an adverse event, this will be brought immediately to the attention of the clinical staff. All participants will be monitored to prevent any possible deficiency or inadequate nutrient and/or caloric intake by means of an evaluation of their dietary intake (dietary recalls and/or food diaries). Participants will also be monitored for injuries or problems associated with increased physical activity.

### Statistical considerations

The present study is designed as a mono-institutional, randomized, controlled four-arm parallel-group trial. The study will be conducted over a 2-year period, during which patients will receive a 6-month intervention, and will be followed until the end of the study period. Study participants will be randomized to one of the four arms: Dietary Intervention (DI); Physical Activity Intervention (PAI); Physical Activity And Dietary Intervention (PADI); or Less Intensive Intervention (LII). The less intensive (or minimal) intervention group will be considered as the control group.

This design is chosen because it has the advantage of testing single or combined interventions, and of comparing the results with the control group (LII).

The mono-institutional study design will be characterized by uniform diagnostic procedures, standard treatment protocols and central pathological evaluation of surgical specimens, thereby limiting variations in the determination of major clinical and pathological factors that determine disease outcome. It will also ensure a uniform follow-up of patients for the study-specific endpoint.

The primary outcome measure will be assessed at the end of the 6-month intervention, by measuring the body weight. We expect a body weight reduction ≥5 % of the baseline body weight in all the intervention arms. The secondary outcome measures will be assessed through the intervention during the follow-up visits: (a) we expect weight control over the study period, measuring the body weight at each follow-up visit; (b) we expect an increase in physical activity levels and reduction in physical inactivity from baseline measured by the pedometer-like devices and from the IPAQ questionnaire. The physical activity target for improving general health is 30 minutes of moderate-to-vigorous intensity every day (equivalent to 10 MET-hours per week) and reducing inactivity; (c) we expect a change in dietary intake and an increased adherence to healthy diet from baseline using the FFQ; (d) we will evaluate the effect of the intervention on long-term breast cancer recurrence; (e) we expect an improvement in quality of life and in health status from baseline using the FACT-B and STAI; (f) we will evaluate the effect of intervention on the blood lipid profile. We expect an increase in HDL cholesterol and a decrease in LDL cholesterol, triglycerides and total cholesterol; (g) we will evaluate the effect of intervention on some biomarkers: estradiol, CRP, insulin, and glucose. Statistical analyses will only be carried out after the end of the trial, with no interim analysis planned.

### Sample size

During 2012, 756 overweight or obese women (with a BMI comprising between 25 and 40 kg/m^2^) underwent surgery for early-stage (non-metastatic) breast cancer at the European Institute of Oncology. Data for these patients were retrieved from our institutional breast cancer patients’ database. The mean weight of these 756 patients was 75 kg and the standard deviation was 9.

The sample size calculation was based on a superiority design of any single intervention (DI, PAI, or PADI) compared to the control group (LII). A sample group of 65 patients (control group) and similar number in any of the three intervention groups (diet and/or exercise) for a total of 195 patients achieve 80 % power to detect a 5 % weight loss (3.75 kg) between the null hypothesis that both group means are 75 kg and the alternative hypothesis that the mean of any of the intervention group 2 is 71.25 after 6 months, with known group standard deviations of 9.0 and 9.0 and with a significance level (alpha) of 0.05 using a two-sided two-sample *t* test. We have foreseen a drop-out of 5 % over the first 6 months.

### Statistical methods

All continuous variables (anthropometric, biomedical, nutritional and psychological measurements) at baseline and at the primary time point (6-month) will be checked for normality and transformed as needed. The Student *t* test, analysis of covariance (ANCOVA) and multiple linear regression models will be used to assess changes in means before and after the intervention. All tests will be two-sided and *P* values <0.05 will be considered statistically significant.

### Dissemination policy

The final report will be presented at the Associazione Italiana per la Ricerca sul Cancro (AIRC). Trial results will be communicated to participants organizing a dedicated event. Results will be also communicated in peer-reviewed journals, in national and international scientific conference, and to the public via press release.

### Data collection, storage and security

All data collected on the questionnaires and all relevant information about the participants will be uploaded on dedicated electronic databases. All forms must be filled in by the researcher or by a person delegated by the researcher. Data will be held according to the International Conference on Harmonisation (ICH) Good Clinical Practice (GCP) E6 Guideline and the Declaration of Helsinki and will be treated with confidentiality, following the current privacy policy [46].

Fully anonymized data from the EPIC food frequency questionnaire will be uploaded on a dedicated online database [47] for the conversion of the FFQ data into a list of food-group and nutrient intake. Data recorded by the pedometer device will be downloaded on a dedicated application [48] adopting security measures to protect personal information [49]. Fully anonymized data will be conveyed periodically to researchers and saved to a password-protected server.

### Registration

The trial is registered with ClinicalTrials.gov, number NCT02622711 (registration date: 2 December 2015) and with ISRCTN registry, number ISRCTN53325751 (registration date: 16 October 2015).

## Discussion

We presented the protocol of a 6-month intervention trial focused on weight loss in a group of overweight or obese women previously treated for early breast cancer. This trial will provide important insights regarding the effect of an intervention carried out in Italy which is designed to improve adherence to a healthy diet and/or to increase physical activity and decrease sedentary time, taking advantage of a pedometer device. Despite the increasing number of trials of weight loss interventions in breast cancer, so far only the DIANA (DIet and ANdrogens) randomized controlled trials have been undertaken on cancer survivors in Italy. They studied the effect of a Mediterranean-macrobiotic lifestyle on breast cancer patients [50] and found a 10 % reduction of testosterone level [50] and an improvement of cardiopulmonary functional capacity and vascular endothelial function following a moderate intensity exercise training combined with the macrobiotic diet [51]. The most recent extension of the DIANA trial [52] is ongoing. It is a two-arm intervention to promote dietary change and a moderate increase in physical activity aimed at decreasing circulating hormone concentrations, breast cancer recurrences, and improving survival.

Our study, with its multi-arm design, has the advantage of evaluating the effect on participants by comparing three different approaches to a common control arm or combining the effect of the interventions. It also appears more appealing to patients because it provides an increased probability of receiving an experimental intervention rather than the control treatment. The educational components of our intervention will be based on national [35] and international recommendations [4], promoting the Mediterranean diet, which includes many dietary features that have been shown to promote health [53]. The scheduled visits and the motivational interviewing approach chosen should ensure a more active role of the patients in lifestyle change to achieve success. Motivational interviewing aims to enhance self-efficacy and personal control for behavior change. It uses an interactive, empathic listening style to increase motivation and confidence, emphasizing the discrepancy between personal goals and health behaviors [54]. Moreover our project will take advantage of a pedometer device to quantify ambulatory activity during walking and running, by means of a common and easily understood metric (i.e., steps). This device is appealing since it objectively monitors physical activity and can be an important means for providing behavioral feedback and motivation. Pedometer-based walking interventions have demonstrated their effectiveness in increasing physical activity in adult populations [55–57]. Objective measuring changes in physical activity in addition to a standard measurement (questionnaire) can add further precision to the physical activity level reached by participants during the intervention.

Due to the increasing concern on the role of obesity in cancer survival and recurrence [11], there is the need for evidence regarding the most effective approach in promoting weight control and the adoption of a healthy lifestyle in breast cancer survivors. The results of this innovative project will provide useful information for future interventions and potentially have a large public health impact on breast cancer survivors.

## Trial status

Recruitment started in November 2015 and is ongoing.

## Abbreviations

BCM, body cell mass; BIVA, bioelectrical impedance vector analysis; BMI, body mass index; CRP, C-reactive protein; DI, dietary intervention; ECW, extracellular water; EPIC, European Prospective Investigation into Cancer and Nutrition; ER, estrogen receptor; FACT-B, Functional Assessment of Cancer Therapy-Breast; FFM, fat-free mass; FFQ, food frequency questionnaire; FM, fat mass; HDL, high-density lipoprotein; HER2, human epidermal growth factor receptor 2; IEO, European Institute of Oncology; IGF, insulin-like growth factor; IPAQ, International Physical Activity Questionnaire; kcal, kilocalorie; LDL, low-density lipoprotein; LII, less intensive intervention; MET, metabolic equivalent; PADI, physical activity and dietary intervention; PAI, physical activity intervention; QoL, quality of life; R, resistance, Ohm; STAI, State-Trait Anxiety Inventory; TBW, total body water; VLDL, very low density lipoprotein; Xc, reactance; Z, vector.
